# Temporal dynamics of photosynthetic inhibition, antioxidant defense, and transcriptomic reprogramming in ‘Crimson Seedless’ grapevine under saline-alkali stress

**DOI:** 10.3389/fpls.2026.1951854

**Published:** 2026-09-24

**Authors:** Chenying Liu, Hao Wang, Kai Gai, Yuzhi Zeng, Songlin Zhang, Chuan Zhang, Jing Zhang, Tengfei Xu, Qiang Jin, Xinyu Wu, Haixia Zhong, Fuchun Zhang, Zhenfan Yu

**Affiliations:** 1 Xinjiang Agricultural University, Urumqi, China; 2 Xinjiang Engineering Research Center for Efficient Cultivation and High-value Utilization of Forest and Fruit Crops, Urumqi, China; 3 Northwest A&F University, Xianyang, China; 4 Institute of Fruits and Vegetables, Academy of Agricultural Sciences of Xinjiang Uyghur Autonomous Region, Urumqi, China; 5 Tarim University, Alar, China

**Keywords:** grapevine, photosynthetic limitation, saline-alkali stress, transcriptional regulation, VvWRKY47

## Abstract

Soil salinization alkalization is among the most critical abiotic stressors affecting crop productivity yet the molecular physiological mechanisms governing grapevine (*Vitis vinifera* L.) responses to combined saline-alkali stress remain poorly understood. This study investigated the temporal dynamics of ‘Crimson Seedless’ grapevine plants propagated from cuttings. Integrated morphological transcriptomic physiological data were collected over a 15-day period of progressive saline-alkali stress. The results showed that saline-alkali stress affected photosynthesis in two phases. During the initial days of stress stomatal closure reduced CO_2_ uptake as evidenced by parallel drops in Pn Gs Ci. In the later stages non-stomatal limitations dominated. Pn decreased by 88% by day 15 while Ci rose despite low Gs. In addition stress triggered ROS accumulation membrane damage osmotic dehydration. Transcriptomic analysis revealed 3,795 differentially expressed genes (DEGs) at 7 days 4,084 at 15 days. Further analysis revealed that stress upregulated MAPK signaling hormone transduction flavonoid biosynthesis while downregulating photosynthesis-related ribosomal genes. Weighted gene co-expression network analysis (WGCNA) identified *VvWRKY47* as a candidate transcription factor. Quantitative real-time PCR (qRT PCR) validation confirmed that *VvWRKY47* expression was induced under saline-alkali conditions. Subcellular localization assays showed the nuclear localization of *VvWRKY47* phenotypic analysis of transgenic Arabidopsis plants overexpressing *VvWRKY47* revealed enhanced growth performance under salt stress compared to empty vector controls.

## 1 Introduction

Soil salinization and alkalization are two of the most severe abiotic stress limiting agricultural productivity worldwide. The impact is even more severe in arid and semi-arid regions (Fan et al., 2026). Reports indicate that approximately 99.13 million hectares of land in China are affected by salinization (Wang et al., 2022; Liu et al., 2026). According to soil characterization, salt stress is often associated with saline (NaCl or Na_2_SO_4_) and alkaline (NaHCO_3_ or Na_2_CO_3_) stress. Saline stress primarily disrupts intracellular ion homeostasis in plant cells. Unlike saline stress, alkaline stress imposes additional pH-induced injury alongside osmotic stress and ion toxicity (Yang et al., 2024; Zhao et al., 2025a). Elucidating plant stress responses and tolerance pathways is critical for remediating salinized soils and ensuring agricultural sustainability. Grapevine (*V. vinifera* L.) represents a particularly relevant model for investigating these stresses. As one of the most economically important fruit crops globally, grapevine is cultivated across diverse climatic zones (Tonietto and Carbonneau, 2004). It is a globally important fruit crop with high economic value. However, most commercial cultivars show moderate to low tolerance to combined salt-alkali stress, suffering irreversible damage to photosynthetic apparatus, root function, and cellular homeostasis. These conditions are not good for growth and development and ultimately severely impact growth, berry quality and yield (Yan et al., 2025). Recent reports suggest that significant part of grape growing areas are facing salinity in irrigation water (Suarez et al., 2019).

Previous studies have established that saline-alkali stress triggers a cascade of physiological dysfunctions in grapevine (Tregeagle et al., 2006) and other crops, including stomatal closure (Downton et al., 1990), diminished chlorophyll content (Foyer and Hanke, 2022), reactive oxygen species (ROS) burst (Ansari et al., 2025), and membrane lipid peroxidation (Kim, 2019) in plants. The Farquhar photosynthesis model provides a robust framework for distinguishing stomatal from non-stomatal constraints on carbon assimilation (Farquhar et al., 1980). Under saline-alkali stress, non-stomatal limitation typically shows reduced Rubisco activity and decreased chlorophyll content (Song et al., 2025). However, these parameters are hardly linked to molecular mechanisms under saline and alkaline stress in grapevine. Similar gap exists in our understanding of antioxidant defense. Some studies reported that SOD (superoxide dismutase), POD (peroxidase), and CAT (catalase) confer partial protection against reactive oxygen species accumulation in many plants against biotic and abiotic stress, the clear dynamic pathways are still not revealed in many plants.

Recent transcriptomic investigations in grapevine have identified many stress-responsive genes, transcription factors (WRKY, EREB, NAC, MYB families), and hormone signaling pathways modulated by individual salt or alkali stresses (Upadhyay et al., 2018). For example, recent study in it was revealed that at early and late stage of stress 52 differentially expressed transcription factors belonging to WRKY, EREB, MYB, NAC, and bHLH families, showed dynamic expression profiles (Upadhyay et al., 2018). Hormone signaling pathways such as abscisic acid (ABA), jasmonic acid (JA), and auxin pathways are extensively reprogrammed under salt stress, with tolerant cultivars exhibiting stronger induction of ABA receptors (PYR/PYL), SnRK2 kinases, and JA-responsive transcription factors (JAZ, MYC2) (Li et al., 2025a). Metabolomic studies have similarly highlighted the accumulation of compatible solutes, phenolic compounds, and secondary metabolites as adaptive responses (Mustapha et al., 2025), while integrated transcriptomic-metabolomic analyses reveal enrichment of flavonoid biosynthesis, phenylpropanoid metabolism, and starch/sucrose pathways in salt-stressed grape roots (Chen et al., 2025). In case of secondary metabolites, salinity promotes accumulation of flavonols, hydroxycinnamic acids, and stilbenes via ROS and ABA-linked activation of the phenylpropanoid pathway (Küskü, 2026). However, these studies have largely examined salt or alkali stress in isolation. Despite these technological advances, molecular mechanisms of grapevine responses to combined saline-alkali stress are not fully understood (Li et al., 2025b; He et al., 2025). Most of the studies were limited to salt or alkali stress in isolation. The synergistic toxicity of mixed salts at high pH likely induces unique transcriptional and metabolic reprogramming distinct from stress alone. The temporal dynamics of the stomatal-to-non-stomatal transition under progressive stress remain uncharacterized in *Vitis*. Some genes were found which were related to saline or alkaline stress, but role of transcription factors is still limited. Recent studies in models crops documented that saline–alkali stress triggered more severe transcriptional down-regulation of photosynthesis-related genes. In grapevine, transcriptomic profiling has similarly revealed that alkali stress exerts more pronounced effects on chloroplast ultrastructure, ion balance, and photosynthetic capacity than salt stress in isolation, yet these studies have examined the two stresses separately rather than in combination (Lu et al., 2022a; Li et al., 2026). Moreover, the regulatory hubs that integrate signal perception with downstream metabolic adaptation remain largely unidentified in grapevine saline-alkali responses. Such hub genes have been characterized as model species. For example, WRKY and NAC transcription factors in Arabidopsis coordinate ROS scavenging with phenylpropanoid biosynthesis under combined stress (Zhu, 2016).

Weighted gene co-expression network analysis (WGCNA) and integrated transcriptome approaches offer powerful frameworks to uncover such regulatory architectures by correlating gene modules with physiological phenotypes and metabolic outputs (Li et al., 2025c). These methods have proven effective in dissecting complex stress responses in alfalfa (Liu et al., 2024), rice (Azad et al., 2024), maize (Wang et al., 2025a), cotton (Hamid et al., 2026) and tomato, yet their application in perennial fruit crops like grapevine under combined saline-alkali stress remains scarce. By integrating time-series transcriptomics with physiological trait measurements, it becomes possible to reconstruct the temporal sequence of molecular events (Yadav et al., 2026) from initial signal transduction (Zhang et al., 2025a) to transcriptional reprogramming (Tian et al., 2026) (Wu et al., 2025). In grapevine specifically, such an approach promises to bridge the current gap and the regulatory logic governing their coordinated deployment under the realistic field condition of combined saline–alkali stress.

The Xinjiang Region of northwest China represents one of the largest grape-producing areas in the country (Yu, 2008; Feng and Xiang-Ling, 2025), yet extensive cultivation occurs on soils characterized by high pH, elevated sodium content, and poor drainage. Under such conditions, plants confront concurrent ion toxicity, osmotic stress, and high-pH-induced nutrient imbalances, collectively termed “saline-alkali stress (Zhao et al., 2023a)” The integrated physiological and molecular mechanisms governing grapevine responses to combined saline-alkali stress remain incompletely understood. The present study was designed to elucidate the physiological and molecular response mechanisms of ‘Crimson Seedless’ grapevine to progressive combined saline-alkali stress through an integrated physiological and transcriptomic strategy. Specifically, this study aimed to characterize the temporal dynamics of photosynthetic inhibition and antioxidant responses under progressive saline-alkali stress, identify key differentially expressed genes and reconstruct co-expression networks, and candidate hub genes and transcription factors. Followed by functional characterization of TFs in model crops. The findings will provide novel insights into grapevine saline-alkali adaptation and to furnish molecular targets for breeding or biotechnological improvement of stress tolerance in this economically vital crop.

## 2 Materials and methods

### 2.1 Experimental materials and treatments

The experiment was conducted in a rain-shelter greenhouse at the Comprehensive Experimental Station of Xinjiang Academy of Agricultural Sciences (43.95°N, 87.48°E). The plant material used was ‘Crimson Seedless’ grape hardwood cuttings, cultivated in plastic pots (upper diameter 23 cm, height 21 cm, bottom diameter 18.5 cm). The culture substrate consisted of a mixture of nutrient medium, vermiculite, and perlite at a ratio of 3:1:1 (v/v/v). Saline-alkali stress treatment was started when the seedlings had developed 10–20 fully expanded leaves. The saline-alkali solution was formulated with NaCl: Na_2_SO_4_:NaHCO_3_:Na_2_CO_3_ at a molar ratio of 1:1:1:1, adjusted to pH 10.0 and electrical conductivity (EC) 2.0 dS·m^-1^ (Dai et al., 2026; Li et al., 2012). Deionized water treatment served as the control. Each treatment comprised three plants with three biological replicates. Leaves were sampled at 0, 3, 5, 7, 10, and 15 days. Physiological and photosynthetic parameters were measured at all time points, while samples for RNA-seq were collected specifically at 3, 7, and 15 days.

### 2.2 Photosynthetic characteristics determination

For each treatment, three uniformly vigorous plants were randomly selected. Fully expanded, healthy functional leaves from the middle-upper canopy at consistent nodal positions were chosen for photosynthetic parameter measurement. The measurement was performed as described in our previous study (Wu et al., 2023; He et al., 2023; Wang et al., 2025b). A portable photosynthesis system (Li-6400XT, LI-COR Biosciences, Lincoln, NE, USA) equipped with an artificial light source leaf chamber was used (He et al., 2023). Measurements were performed between 10:00 and 12:30 h, with the leaf chamber temperature maintained consistent with ambient temperature. Photosynthetically active radiation (PAR) was set at ten gradients: 0, 75, 150, 300, 500, 750, 1000, 1500, 2000, and 2500 μmol·m^-^²·s^-1^. Light response curves were fitted using the modified rectangular hyperbola model to calculate dark respiration rate (*R*_D_), maximum net photosynthetic rate (*P_n_*), light saturation point (*I_sat_*), light compensation point (*I_C_*), and apparent quantum yield (AQY). Stomatal limitation (*Ls*) was calculated as *Ls* = 1 - (*Ci*/*Ca*), where *Ca* (ambient CO_2_ concentration) was maintained at 400 µmol mol^-1^ during measurements, and *Ci* (intercellular CO_2_ concentration) was recorded by the Li-6400XT system. The *Ci/Ca* ratio was also calculated to assess the relative contribution of stomatal vs. non-stomatal limitations.

### 2.3 Physiological and biochemical index determination

MDA content was determined by the thiobarbituric acid (TBA) method with modifications (Heath and Packer, 1968). Briefly, 0.5 g leaf samples were homogenized in 6 mL 10% TCA, centrifuged, and 3 mL supernatant was mixed with 3 mL 0.6% TBA. After boiling for 30 min and cooling, absorbance was read at 450, 532, and 600 nm. MDA concentration was calculated as: MDA (μmol/L) = 6.45 × (OD_532_ − OD_600_) − 0.56 × OD_450_ (Velikova et al., 2000). Antioxidant enzyme activities were assayed spectrophotometrically using commercial kits. Catalase (CAT; EC 1.11.1.6), peroxidase (POD; EC 1.11.1.7), and superoxide dismutase (SOD; EC 1.15.1.1) activities were determined using commercial assay kits (Suzhou Comin Biotechnology Co., Ltd., Suzhou, China; http://www.cominbio.com) according to the manufacturer’s instructions. H_2_O_2_ content was measured using a hydrogen peroxide detection kit (Shanghai Enzyme-linked Biotechnology, Shanghai, China http://www.mlbio.cn/). O_2_^-^ scavenging capacity was assayed with a superoxide anion scavenging capacity kit.

Root vigor was measured by the triphenyltetrazolium chloride (TTC) method using a commercial kit (Solarbio, Beijing, China https://www.solarbio.com/). Relative water content (RWC) was measured following (Barrs and Weatherley, 1962) with modifications. The 3rd to 4th mature functional leaves were sampled, weighed for fresh weight (Wf), immersed in deionized water for 8–12 h for turgid weight (Wt), then oven-dried at 80 °C to constant weight for dry weight (Wd). RWC (%) = [(Wf − Wd)/(Wt − Wd)] × 100 (Flexas et al., 2002).

### 2.4 Transcriptome sequencing and data analysis

Total RNA was extracted from leaf tissues using a polysaccharide- and polyphenol-specific plant total RNA extraction kit (TIANGEN Biotech, Beijing, China). RNA concentration and purity were assessed using a NanoDrop 2000 spectrophotometer (Thermo Fisher Scientific, Waltham, MA, USA), and RNA integrity was evaluated on an Agilent 2100 Bioanalyzer (Agilent Technologies, Santa Clara, CA, USA) with RIN values > 7.0. For qualified samples, mRNA by oligo(dT) bead selection, fragmented, and used for cDNA library construction following the TruSeq RNA Library Prep protocol (Illumina). Paired-end sequencing (2 × 150 bp) was performed on the Illumina NovaSeq 6000 platform (Illumina, San Diego, CA, USA). Raw reads were filtered using fastp (v0.19.6 or later) to remove adapter sequences, trim low-quality bases, and discard reads shorter than 50 bp, yielding clean data (Chen et al., 2018). Clean reads were mapped to the *V. vinifera* reference genome (PN40024.T2T.v5 available at https://grapedia.org/genomes/) (Velt et al., 2023) using HISAT2 (v2.1.0 or later) with default parameters (Kim et al., 2015). Gene expression levels were quantified as fragments per kilobase of transcript per million mapped reads (FPKM) using StringTie (v1.3.4d or later; (Pertea et al., 2015)). Differentially expressed genes (DEGs) were identified using DESeq2 (v1.22.1 or later) with a false discovery rate (FDR) < 0.05 and |log_2_ fold change| ≥ 1 (Love et al., 2014), followed by Gene Ontology (GO) functional annotation and Kyoto Encyclopedia of Genes and Genomes (KEGG) pathway enrichment analysis. Details are provided in **Supplementary Table S1** (quality summary of RNA seq data), S2 (DEGs) and S3 (GO and KEGG).

### 2.5 Gene set enrichment analysis (GSEA)

Gene Set Enrichment Analysis (GSEA) was performed using the `clusterProfiler` R package (v4.4.4) with KEGG gene sets to identify globally enriched pathways, with significance determined by a false discovery rate (FDR) < 0.05 and normalized enrichment score (|NES|) > 1 (Zhang and Parkin, 2025). Genes were ranked by absolute Pearson correlation coefficient, and the top pairs were visualized in a correlation heatmap using the `pheatmap` package (v1.0.12) in R (Zhao et al., 2025c). Shared KEGG pathways supported by both differentially expressed genes (DEGs) were identified by pathway co-annotation as joint evidence for integrated biological interpretation followed by previously described method (Yadav et al., 2026). Selected key differential genes were validated by quantitative real-time PCR (qRT-PCR) using SYBR Green fluorescent dye and gene-specific primers to verify the accuracy of transcriptome sequencing results. The methods are previously described in our previous research (Yadav et al., 2025; Wang et al., 2024a). To elucidate the molecular regulatory mechanisms underlying physiological and biochemical phenotypic changes under saline-alkali stress, a weighted gene co-expression network was constructed using the WGCNA R package (v1.72.1) (Fan et al., 2024). The details of genes are provided in **Supplementary Table S4** (GESA), cluster genes (**Supplementary Table S5**) and hub genes in (**Supplementary Table S6**).

### 2.6 Subcellular localization assays and functional validation in Arabidopsis OE plants

The coding sequence of *VvWRKY47* was cloned into pC2300-GFP to produce a 35S:*VvWRKY47-*GFP fusion. The construct and empty vector were transformed into *Agrobacterium tumefaciens* GV3101 and infiltrated into *Nicotiana benthamiana* leaves as described in our previous research (Zhang et al., 2025c). After 48 h at 24 °C, GFP and mCherry signals were captured on a Carl Zeiss LSM 510 confocal microscope. Four different images of mCherry for nuclei, bright-field for cell morphology, GFP for WRKY47 localization, and Merge for composite overlay were captured (Xu et al., 2025).

For functional characterization OE plants were generated. *VvWRKY47* CDS (stop codon removed) was PCR-amplified and cloned into a recombinant vector (ClonExpress II, Vazyme Biotech). The construct was transformed into *E. coli* DH5α, then into *A. tumefaciens* GV3101 by freeze-thaw. Arabidopsis Col-0 plants were transformed via floral dip followed by our previous published protocols (Xu et al., 2025). T1 seedlings were selected on 1/2 MS + 75 mg/L kanamycin. T3 homozygous lines were confirmed by qRT-PCR. Vernalized seeds were sown on two types of MS agar plates. Standard MS medium (control) and MS medium supplemented with saline-alkaline stress were used for experiment. The plates were placed under controlled growth conditions (23 °C, 16 h light/8 h dark photoperiod) for growth. Seedling growth was monitored regularly, and phenotypic images were captured once distinct differences in growth became apparent between the groups. Following imaging, rosette leaves were harvested (n=10) and immediately stored at −80 °C for subsequent analysis. The list of primers is provided in **Supplementary Table S7**.

### 2.7 Statistical analyses

Physiological parameters were monitored at 0, 3, 5, 7, 10, and 15 days of saline-alkali stress, with mock-treated plants (CK) sampled at identical intervals. Net photosynthetic rate (A), stomatal conductance (*gs*), intercellular CO_2_ concentration (*Ci*), transpiration rate (E), water use efficiency (WUE), malondialdehyde (MDA), H_2_O_2_, O_2_^-^, superoxide dismutase (SOD), peroxidase (POD), catalase (CAT), root vigor, and relative water content (RWC, %) were quantified. Data were compiled in Excel 2019 and analyzed by one-way ANOVA at each time point. Line graphs plotting stress versus CK across the time course were generated using Python scripts (Matplotlib v3.7.2, Seaborn v0.12.2). Final values are mean ± SD; n=3 biological replicates per treatment per time point.

## 3 Results

### 3.1 Effects of saline-alkali stress on the phenotype and photosynthetic characteristics of crimson seedless grape seedlings

Saline-alkali treatment significantly inhibited the normal growth of ‘Crimson Seedless’ grape seedlings, with stress symptoms intensifying progressively as the treatment duration extended. At 7 days post-treatment (dpt), growth of the saline-alkali stress group was initially suppressed. Observation revealed that chlorosis began to appear at the margins of middle and lower leaves, with some older leaves showed brownish necrosis at the leaf tips (**Figure 1A**). New shoot growth was markedly slower than that of the control group, displaying preliminary characteristics of salt and alkali injury. At 15 dpt, stress symptoms were further exacerbated. Leaves in the treatment group developed extensive chlorosis and necrotic spots, with marginal scorch extending toward the leaf veins. In contrast, control plants (CK) maintained normal growth status with dark green foliage. These results indicate that sustained saline-alkali stress caused irreversible morphological damage to ‘Crimson Seedless’ seedlings (**Figure 1**).

**Figure 1:**
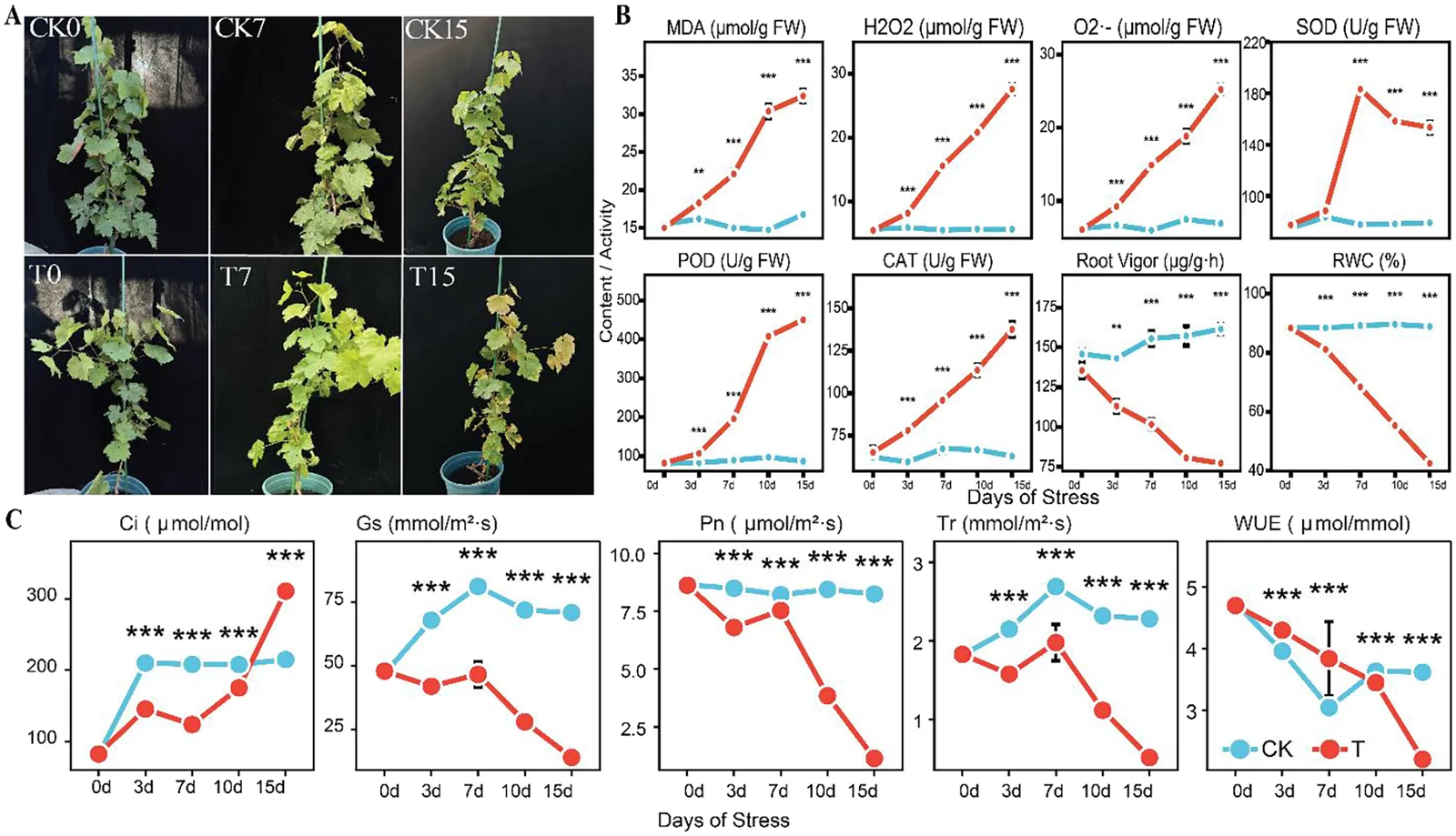
Physiological and photosynthetic responses of ‘Crimson Seedless’. **(A)** Phenotypic changes of seedlings under treatment (T) and control (CK) at 0, 3, 7, 10 and 15 d. **(B)** Time-course dynamics of oxidative stress markers, antioxidant enzyme activities, root vigor, and RWC (%). **(C)** Photosynthetic gas exchange parameters. Data are mean ± SE (n ≥ 3). **, *** indicate significant differences at p < 0.01, and p < 0.001, respectively.

### 3.2 Effects of saline-alkali stress on physiological, biochemical and photosynthetic characteristics

Under saline-alkali stress, plants showed a pronounced oxidative stress response accompanied by severe osmotic dehydration, with massive accumulation of reactive oxygen species (ROS) and membrane lipid peroxidation (**Figure 1B**). Saline-alkali treatment induced rapid and sustained production of H_2_O_2_ and ·O_2_^-^, both of which exhibited significant linear upward trends with increasing stress duration (**Figure 1B**). MDA, an indicator of membrane lipid peroxidation, also showed an extremely significant and sustained increase. To neutralize the oxidative damage, SOD, functioning as the first line of defense, displayed a typical stress-response pattern. SOD activity peaked at approximately 7 d and subsequently declined. POD and CAT, primarily responsible for eliminating downstream H_2_O_2_, maintained sustained and extremely significant increases in activity throughout the 15-day period in response to the continuously accumulating hydrogen peroxide (**Figure 1B**). The saline-alkali environment induced severe drought-like conditions, which resulted in a precipitous decline in leaf relative water content (RWC) over time. Additionally, root vigor in the treatment group decreased significantly from 3 d onward, falling to markedly lower levels than the control by 15 d.

To investigate the physiological mechanisms underlying the phenotypic damage, leaf photosynthetic parameters were determined at different time points (**Figure 1C**). At 0 dpt, no significant difference in net photosynthetic rate (*Pn*) was observed among groups. As stress progressed, *Pn* declined significantly. At 3 dpt, *Pn* decreased to approximately 7.0 μmol·m^-^²·s^-1^, which was significantly lower than that of the control group. By 15 dpt, *Pn* was merely approximately 1.0 μmol·m^-^²·s^-1^, representing a reduction of approximately 88% relative to the control. During the early stress phase (3–7 dpt), stomatal conductance (*Gs*) in the treatment group decreased significantly, accompanied by a concurrent reduction in intercellular CO_2_ concentration (*Ci*). *Gs* remained at extremely low levels, whereas *Ci* was paradoxically elevated to approximately 310 μmol·mol^-1^ during late stress phase. Concurrently, transpiration rate (*Tr*) in the treatment group declined sharply after 7 dpt, reaching near-zero values by 15 dpt. In addition, we also calculated stomatal limitation (*Ls*) and *Ci/Ca* ratios. At 3 days post-treatment (dpt), *Ci/Ca* decreased from 0.554 ± 0.021 in controls to 0.413 ± 0.023 in treated plants, while Ls increased from 0.446 ± 0.021 to 0.588 ± 0.023 (P < 0.05). By 7 dpt, *Ls* reached its maximum value in treated plants vs. in controls (P < 0.01). However, at 15 dpt, *Ls* in treated plants declined to 0.528 ± 0.029 while *Ci/Ca* increased from 0.396 at 7 dpt to 0.472 at 15 dpt (P < 0.05). WUE initially decreased at 3 dpt, then showed a transient recovery at 7 dpt, followed by a significant decline at 10 and 15 dpt as photosynthesis collapsed more severely than transpiration. Combined with the yellowing and wilting of leaves following saline-alkali stress, the leaf tissues suffered physical damage from high pH and ion toxicity, leading to reduced Rubisco enzyme activity or impaired electron transport chain function.

### 3.3 Transcriptome differential expression analysis

To investigate the effects of saline-alkali stress on gene expression in ‘Crimson Seedless’ grape at the transcriptional level, transcriptome sequencing was performed on samples collected at 3 d (CK3/T3), 7 d (CK7/T7), and 15 d (CK15/T15) post-treatment. RNA-seq was performed on 18 samples (3 biological replicates per group: CK3, T3, CK7, T7, CK15, T15). A total of ~93.6–94.9 million raw reads were generated per sample, yielding ~7.0–9.5 Gb of raw bases (**Supplementary Table 1**). After quality filtering, clean reads ranged from 44.9 to 63.2 million per sample with a clean ratio of 99.98%. The Q20 and Q30 values exceeded 99.35% and 97.65%, respectively, and GC content was approximately 45.9–46.2%, showing high-quality sequencing data suitable for downstream differential expression analysis (**Supplementary Table 1**). Hierarchical cluster analysis (HCA) and principal component analysis (PCA) were conducted on gene expression profiles across all samples (**Figure 2A**). As shown in **Figure 2B**, the dendrogram revealed that three biological replicates from the same point and treatment clustered tightly together, indicating high experimental reproducibility. With prolonged stress duration (from 3 d to 15 d), the distance between treatment and control groups progressively increased in the PCA plot, suggesting that the impact of saline-alkali stress on gene expression patterns was significantly intensified over time.

**Figure 2:**
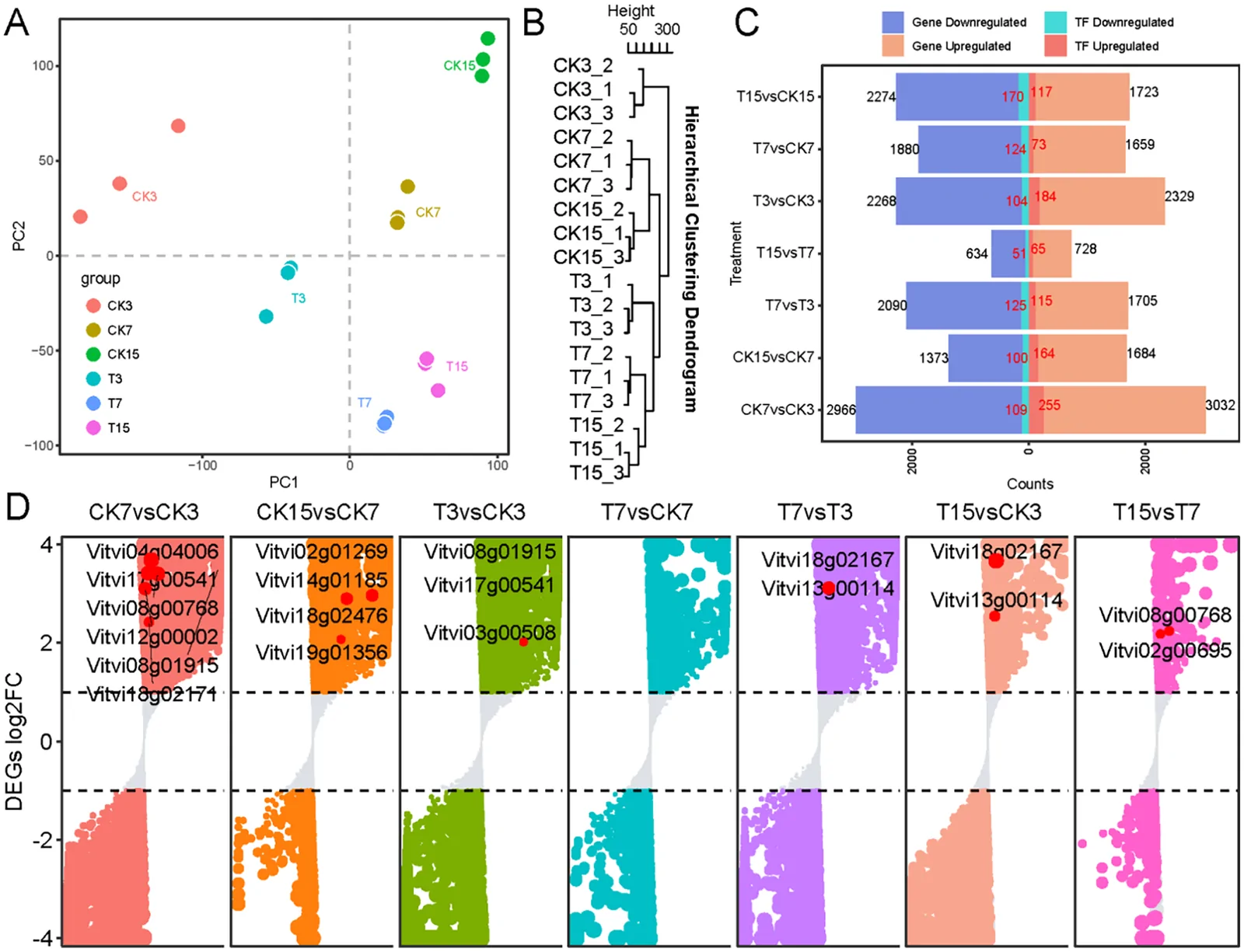
Transcriptome profiling of ‘Crimson Seedless’ under saline-alkali stress. **(A)** PCA of different samples. **(B)** Hierarchical clustering dendrogram of all samples. **(C)** Numbers of up- and down-regulated genes and transcription factors. **(D)** Upset volcano plots of DEGs for pairwise comparisons.

To identify key genes responsive to saline-alkali stress, pairwise comparisons were performed between different groups. As illustrated in **Figure 2C**, the number of differentially expressed genes (DEGs) varied markedly among comparison groups (**Supplementary Table 2**). At 7 d post-treatment (T7 vs. CK7), a total of 3,795 DEGs were identified (1,705 up-regulated and 2,090 down-regulated), whereas at 15 d post-treatment (T15 vs. CK15), the total number of DEGs increased to 4,084. This shows that transcriptional reprogramming became more extensive as stress persisted. Further, transcriptome data was used to find transcription factors. In the T3 vs. CK3 comparison, the number of differentially expressed TFs was relatively limited (184 up-regulated and 104 down-regulated). However, in the T15 vs. CK15 comparison, the numbers of up-regulated and down-regulated TFs reached 170 and 117, respectively, showing that transcriptional regulatory networks played a critical role during the late stress phase (**Figure 2C**). To further screen for core responsive genes, volcano plots were employed to visualize DEGs across different comparison groups (**Figure 2D**). Results showed that genes shared by multiple comparison groups may serve central roles in saline-alkali stress adaptation. For instance, *Vitvi08g01915* and *Vitvi17g00541* exhibited expression alterations across multiple time points during the stress response (**Figure 2D**). Notably, *Vitvi18g02167* and *Vitvi13g00114* were identified as strongly up-regulated in both T7 vs. T3 and T15 vs. CK15 comparisons, suggesting their potential involvement in the progressive stress response. Additionally, *Vitvi04g04006* and *Vitvi08g00768* were among the most prominently down-regulated genes in the CK7 vs. CK3 comparison, while *Vitvi18g02167* and *Vitvi13g00114* showed marked up-regulation in T15 vs. CK15 and T15 vs. T7 comparisons, respectively. The validity of RNA-seq data was correlation coefficient plot comparing RNA-seq and qRT-PCR fold changes (**Supplementary Figure S1**).

### 3.4 GO and KEGG enrichment analysis of differentially expressed genes

To elucidate the molecular mechanisms underlying the response of ‘Crimson Seedless’ grape to saline-alkali stress, functional annotation and enrichment analyses were performed on differentially expressed genes (**Supplementary Table 3**). GO enrichment analysis (**Figure 3**) revealed that in T7 vs. CK7 and T15 vs. CK15, up-regulated genes were predominantly enriched in biological processes including response to salt stress, response to reactive oxygen species, response to hydrogen peroxide, response to heat, and protein folding. Conversely, down-regulated genes in these comparisons were concentrated in cell wall organization, cell wall macromolecule catabolic process, protein refolding, and chloroplast RNA processing. In T3 vs. CK3, early stress responses were marked by enrichment in protein refolding and response to heat, while T15 vs. T7 showed progressive intensification of carbohydrate metabolic process and response to reactive oxygen species.

**Figure 3:**
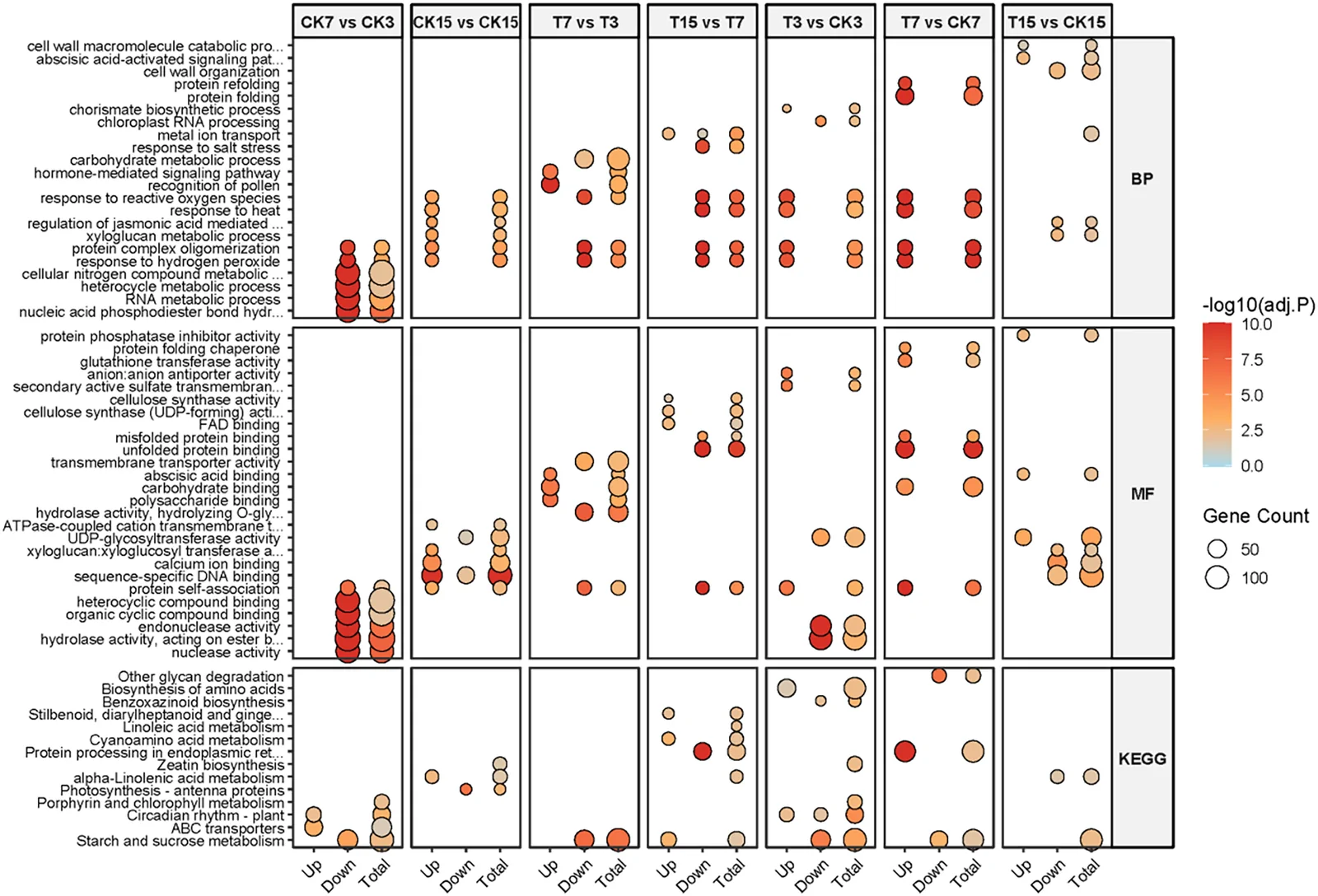
Functional annotation and pathway enrichment of DEGs. GO enrichment analysis of up-regulated, down-regulated, and total DEGs across biological process (BP), molecular function (MF), and KEGG pathway enrichment of up-regulated, down-regulated, and total DEGs. Bubble size reflects gene count; color intensity corresponds to −log_10_(adjusted P-value).

KEGG pathway enrichment analysis (**Figure 3**; **Supplementary Table 3**) further revealed the reprogramming strategies of cellular metabolic networks. Up-regulated genes in T7 vs. CK7 and T15 vs. CK15 were significantly enriched in protein processing in the endoplasmic reticulum, starch and sucrose metabolism, and alpha-linolenic acid metabolism, reflecting osmolyte accumulation and membrane lipid remodeling. In contrast, photosynthesis-antenna proteins, porphyrin and chlorophyll metabolism, and zeatin biosynthesis were significantly suppressed. Notably, linoleic acid metabolism and ABC transporters also emerged as enriched pathways in stress-responsive transcriptome reprogramming, particularly in T15 vs. CK15 and T7 vs. CK7. Additionally, molecular function enrichment revealed significant terms including protein folding chaperone activity, glutathione transferase activity, anion:anion antiporter activity, and transmembrane transporter activity, reflecting enhanced protein stabilization and ion homeostasis mechanisms under saline-alkali stress.

### 3.5 Gene set enrichment analysis (GSEA)

GSEA was further used to identify the global expression trends of metabolic pathways under saline-alkali stress. The GSEA heatmap (**Figure 4A**) revealed that the activation and suppression patterns of pathways became increasingly pronounced with prolonged stress duration, particularly from T7 vs. CK7 to T15 vs. CK15 (**Supplementary Table 4**). Growth-related fundamental metabolic pathways, including `Photosynthesis-antenna proteins`, `Ribosome`, `Pyrimidine metabolism`, and `Pentose and glucuronate interconversions`, showed a consistent and significant downward trend. In contrast, stress resistance-associated pathways displayed significant up-regulation, encompassing `MAPK signaling pathway`, `Plant hormone signal transduction`, `Flavonoid biosynthesis`, `Starch and sucrose metabolism`, `Glutathione metabolism`, `Glyoxylate and dicarboxylate metabolism`, and `ABC transporters`. In addition, `Plant-pathogen interaction` and `Homologous recombination` also showed enrichment in certain comparisons, suggesting cross-talk between abiotic and biotic stress responses. Further analysis showed, `Biosynthesis of amino acids` and `Aminoacyl-tRNA biosynthesis” were suppressed in later stress stages.

**Figure 4:**
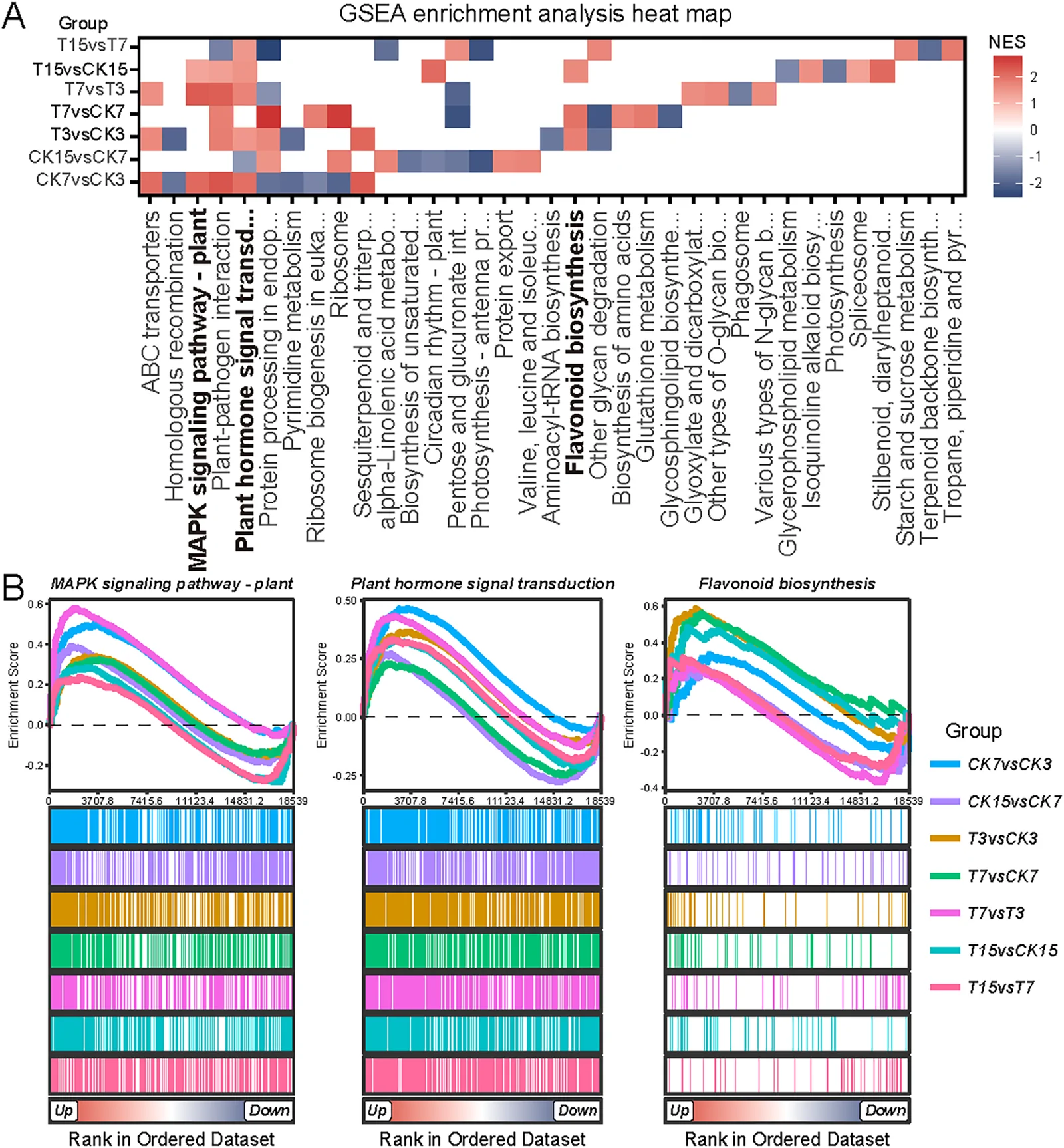
GSEA enrichment analysis of ‘Crimson Seedless’ under saline-alkali stress. **(A)** Heat map of normalized enrichment scores (NES) across pairwise comparisons. **(B)** Enrichment score curves for MAPK signaling, plant hormone signal transduction, and flavonoid biosynthesis.

The enrichment analysis of three core pathways (**Figure 4B**) showed that the enrichment score curves for `MAPK signaling pathway-plant`, `Plant hormone signal transduction`, and `Flavonoid biosynthesis` all peaked at the left side of the ranked gene list, with particularly strong manifestations in stress-treated groups (T7 vs. CK7 and T15 vs. CK15). These results suggest that upon perception of saline-alkali stress, ‘Crimson Seedless’ grape rapidly transmits stress signals through MAPK cascade reactions and hormone signaling networks, subsequently inducing massive expression of genes involved in the synthesis of secondary metabolites such as flavonoids. Through the accumulation of antioxidant substances to scavenge reactive oxygen species, the plant constructs a systematic defense mechanism spanning from `signal perception` to `metabolic adaptation`.

### 3.6 Clustering analysis and functional enrichment of differential gene expression patterns

To further investigate the temporal dynamic characteristics of gene expression, all differentially expressed genes were partitioned into eight distinct clusters (Clusters C1–C8) with specific expression patterns through clustering analysis (**Supplementary Table 5**). As shown in **Figure 5**, genes in Clusters C4 and C5 exhibited a sustained and significant downward trend in expression under saline-alkali stress. Functional enrichment analysis revealed that these two clusters were predominantly associated with `photosynthesis`, `chloroplast organization`, and `porphyrin and chlorophyll metabolism`. These results transcriptionally showed that saline-alkali stress suppressed the expression of chloroplast structural components and photosynthesis-related enzyme genes. Furthermore, genes in Cluster C1 displayed relatively high expression during the initial stress phase but subsequently declined and were enriched in `protein folding` and `protein processing in endoplasmic reticulum` pathways. Conversely, genes in Clusters C7 and C8 showed significant up-regulation during the late stress phase (T15), representing late-stage adaptive responses of the plant. Notably, Cluster C7 was significantly enriched in `Proteasome`, `ubiquitin-mediated proteolysis`, and `glutathione metabolism` pathways. Meanwhile, the enrichment of `carotenoid biosynthetic process` and `Circadian rhythm-plant` was observed in Cluster C8 (**Supplementary Table 5**).

**Figure 5:**
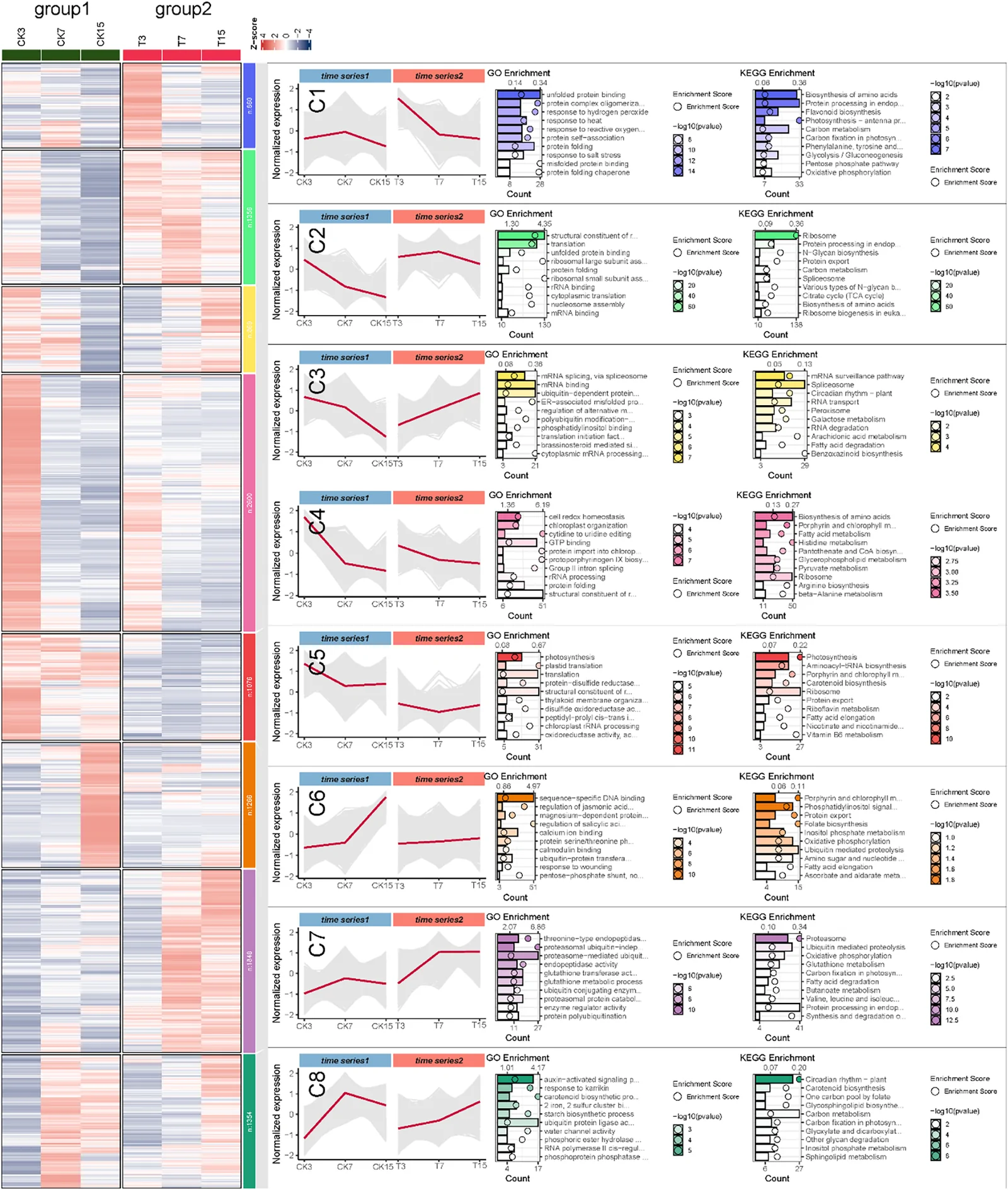
Cluster analysis of DEGs. Group 1 denotes heatmap of CK treatments while group 2 denotes heatmap of treated groups. Different clusters were identified and GO and KEGG analysis were performed.

### 3.7 Weighted gene co-expression network analysis (WGCNA) and module-trait association

To further elucidate the potential molecular regulatory mechanisms, we constructed a weighted gene co-expression network (WGCNA) using transcriptome data and performed integrated analysis with previously determined photosynthetic parameters and physiological-biochemical indices (**Figure 6A**, **Supplementary Table 6**). Through systematic hierarchical clustering, genes with similar expression patterns were successfully separated into multiple co-expression modules (**Figure 6A**). Module-trait relationship analysis revealed a high degree of coordination between specific gene clusters and phenotypic variations under saline-alkali stress. Notably, the `deepskyblue` and `coral3` module showed the most significant co-expression modules (**Supplementary Table 6**). The detailed analysis of GO and KEGG enrichment of key modules was also performed in top four significant modules (**Figure 6B**). To key hubs regulating this stress-responsive pathway, gene interaction relationships within the critical module were extracted and a co-expression regulatory network was constructed (**Figure 6C**). Network topological structure analysis revealed inter-module crosstalk between the yellow module and its adjacent modules. Within the core regulatory network of the yellow module, node weights were calculated based on gene connectivity (degree centrality), ultimately identifying a core gene (hub gene) positioned at the absolute topological center of the network-*Vitvi07g00523* (**Figure 6C**). Functional annotation indicated that this gene encodes a WRKY family transcription factor, WRKY47. As the hub of this core stress-resistance network, *Vitvi07g00523* (WRKY47) most likely functions as a key candidate transcription factor for upstream transcriptional regulation. The heatmap showed elevated expression the *Vitvi07g00523* (WRKY47) in all treated groups (**Figure 6D**).

**Figure 6:**
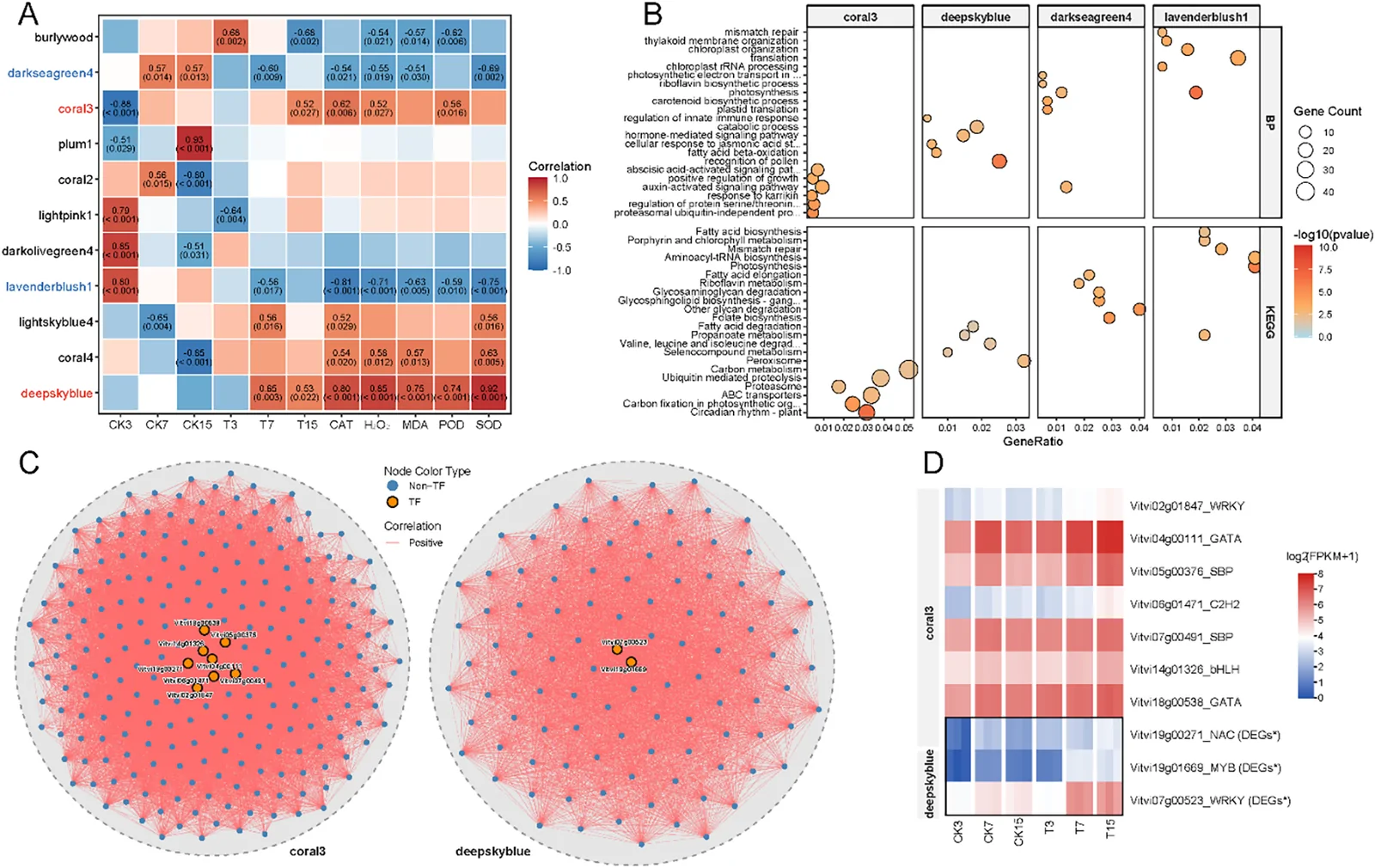
Weighted gene co-expression network analysis (WGCNA) and hub gene identification. **(A)** Module-trait correlation heat map. Colors represent Pearson correlation coefficients between module eigengenes and physiological traits. **(B)** GO and KEGG enrichment of key modules (coral3, deep sky blue, dark sea green 4, lavender blush 1). Bubble size: gene count; color: −log_10_(p-value). **(C)** Co-expression networks of coral3 (left) and deep sky blue (right) modules. **(D)** Expression heat map of hub TFs across samples.

### 3.8 Subcellular localization and functional characterization in model plants

WRKY transcription factors identified from WGCNA were further subjected to qRT-PCR analysis. The results revealed that the expression level of *Vitvi07g00523* (*WRKY47*) was higher in stressed plants than in control (CK) ‘Crimson Seedless’ grapevine. Further detailed analysis indicated that *VvWRKY47* expression was induced several-fold following 7 and 15 days of exposure to saline and alkaline conditions in grape (**Figure 7A**). To further elucidate the functional properties of the candidate protein, subcellular localization was assessed. The coding sequence of *VvWRKY47*, identified as a hub gene from WGCNA, was cloned from leaf cDNA of saline-alkali-treated ‘Crimson Seedless’ grapevine. Transient expression of a *VvWRKY47*-GFP fusion in tobacco epidermal cells demonstrated strict nuclear localization, as confirmed by co-localization with the mCherry nuclear marker (**Figure 7B**). Relative expression analysis in overexpression (OE) lines showed that *VvWRKY47* was highly expressed in transgenic Arabidopsis plants (**Figure 7C**). The phenotypic response of *VvWRKY47*-overexpressing Arabidopsis to salt and alkaline stress was also examined. Empty vector (EV) and T3 overexpression (OE) lines were grown on MS medium and subsequently transferred to control (CK) or stress media. Observations indicated that OE plants performed better than EV plants under stress conditions, whereas both showed similar growth trajectories under control conditions (**Figure 7D**).

**Figure 7:**
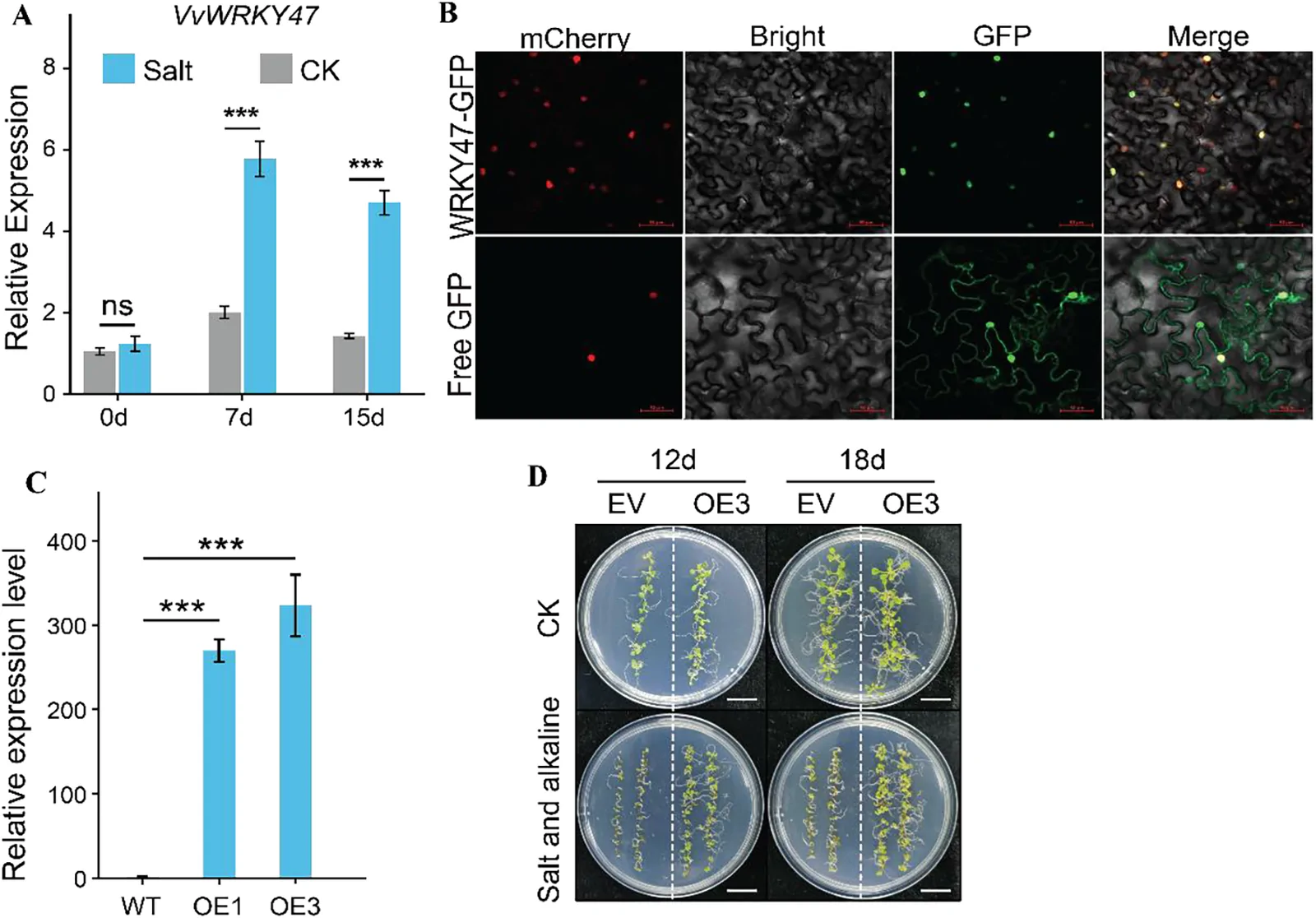
Subcellular localization and salt stress tolerance of *VvWRKY47*. **(A)** Relative expression profiling *VvWRKY47* in ‘Crimson Seedless’ grapevine. **(B)** Subcellular localization of *VvWRKY47* in *N. benthamiana* epidermal cells. **(C)** Expression profiling of *VvWRKY47* in transgenic Arabidopsis plants. **(D)** Phenotypic response of *VvWRKY47*-overexpressing Arabidopsis to salt stress. *** indicate significant differences at p < 0.001.

## 4 Discussion

### 4.1 Temporal dynamics of photosynthetic inhibition

Most of the plants suffer largely due to saline-alkaline stress and growth and development severely disrupted. Previous findings showed that such stress significantly impact growth and development of grapevine. For instance, authors found that vine shoots struggled to develop in ‘Red Globe’ grapes due to saline and alkaline stress (Wang et al., 2018). In our research leaves showed initial marginal chlorosis of older leaves followed by tip necrosis. Similar findings were reported in grapevine rootstocks under salinity (Hakeem et al., 2025; Rius-Garcia et al., 2025). Moreover, Zhao et al., also reported that ‘Cabernet Sauvignon’ shows marginal necrosis under saline-alkali stress (Zhao et al., 2023b). Our data also showed that at later stages exposure to saline-alkaline stress develops some irreversible changes in leaves and accelerates chlorophyll degradation. The findings showed that such observations were observed in other grapevines also (Lu et al., 2022c; Lai et al., 2025). In addition, in some grapevine rootstocks such as ‘3309M’ and ‘Kangzhen’ suppression of new shoot growth was observed (Hakeem et al., 2025).

Oxidative stress is a common response in plants exposed to various abiotic and biotic stresses. Accumulation of ROS and the parallel rise in MDA content indicate severe membrane lipid peroxidation under saline-alkali stress (Lai et al., 2025). Our research results supported this theory and our results showed that SOD activity peaked at 7 dpt before declining, while POD and CAT sustained their upward trajectory through 15 dpt. The possible mechanism could be related to high concentrations of H_2_O_2_ and O_2_^-^. This decline in SOD activity may be attributed to the saturation of its O_2_^-^ substrate as the system reaches oxidative equilibrium under high concentrations of H_2_O_2_ and O_2_^-^. Some studies also showed that SOD as the first line of defense responds rapidly but transiently, while POD and CAT represent secondary, sustained scavenging systems that are transcriptionally up regulated. This differential pattern shows the hierarchical organization of the antioxidant system (Iqbal et al., 2026; Wang et al., 2024b). Moreover, pattern of SOD changes shows that rate of ·O_2_^-^ generation exceeded the enzyme’s catalytic capacity, leading to substrate-induced inhibition. Similar phenomenon was documented by researchers in other grape varieties (Lu et al., 2022b; Zu-Man et al., 2024). ‘Cabernet Sauvignon’ plants showed imbalance between ROS production and scavenging capacity. Similar findings were reported in some other varieties and it seems this phenomenon is related saline-alkali stress in grapevine (Song et al., 2024).

Saline-Alkaline stressed plants showed decline in photosynthetic capacity of ‘Crimson Seedless’ grapevine. The severity intensified from 3 to 15 days post treatment. At 3 dpt, the *Pn* was significantly lower in the treated groups compared to the control. As the stress progressed, *Pn* continued to decline, resulting in increasingly pronounced differences between the treated and control groups over time. Photosynthesis activities were quite normal in CK groups at different time periods, whereas significant differences were seen in treated groups. The temporal trajectory aligns with the two-phase stress response model described in earlier research (Schubert et al., 2009; Sümer et al., 2004). In addition, during the early phase (3 dpt), the parallel decline in stomatal conductance (*Gs*) and intercellular CO_2_ concentration (*Ci*) indicated that stomatal closure played important role. By 15 dpt, Gs remained at extremely low levels while *Ci* was paradoxically elevated, indicating a shift from stomatal to non-stomatal limitation. Similar results were shown in research that ABA-mediated guard cell signaling under osmotic stress (Hsu et al., 2021). Stomatal response shows an adaptive mechanism to reduce transpirational water loss under saline-alkali conditions (Harrison et al., 2020). Morphological observation in our results showed that saline-alkali stress leads to yellowing and wilting of leaves continuously exposed for two weeks. Similar findings were reported by Zhang et al., 2023 where alkali stress exacerbates chloroplast swelling and thylakoid disorganization (Zhang et al., 2023). Overall, saline-alkali stress induced a cascade of deleterious effects in ‘Crimson Seedless’ grape seedlings. This started with osmotic stress and stomatal closure at 3 dpt, progressing through ROS-mediated oxidative damage and antioxidant system reorganization, and culminating by 15 dpt in irreversible photosynthetic inhibition dominated by non-stomatal limitations.

### 4.2 *WRKY47* as a central hub integrating stress signaling

Our data revealed dynamic changes in genes and transcription factors. An independent study on grapes also revealed similar findings, showing progressive changes in transcriptome profiling throughout salinity stress (Gajjar et al., 2024). Recent research in many crops has shown that WGCNA is a promising tool for candidate gene selection. For instance, in cotton (Zhao et al., 2025b), Chinese sea bass (Zhu et al., 2023) and soybean (Liu et al., 2025). WGCNA-based modules revealed critical hub genes related to saline-alkaline stress. WGCNA identified genes from the JA signaling pathway, particularly the key transcription factor MsMYC2 (Myelocytomatosis protein 2), as a pivotal contributor to saline-alkaline tolerance (Zhang et al., 2025b). During signal transduction in response to salt-alkali stress, transcription factors serve as a bridge between stimulus signals and associated genes. Major transcription factor families such as AP2/ERF, NAC, HD-Zip/bZIP, MYC/MYB, WRKY, and bHLH have been identified and characterized in many crops (Zhang et al., 2021; Shah et al., 2021). The WRKY gene family, as a plant-specific transcription factor group, plays important roles in various response pathways to saline and alkali stresses (Li et al., 2020). Some WRKY genes were characterized in some crops. For instance, *ZmWRKY114* in maize (Bo et al., 2020), SbWRKY50 in sweet sorghum (Song et al., 2020), *MxWRKY64* in *A. thaliana* (Han et al., 2021)*, MdWRKY100* in *M. domestica* (Ma et al., 2021) and *GmWRKY16* in soybean reveled role of these TFs in saline-alkaline stress (Ma et al., 2021). Our WGCNA results identified *VvWRKY47* as a central hub coordinating saline-alkali stress responses in grapevine. High member module membership and degree centrality establish *VvWRKY47* as the topological key gene. Moreover, gene expression and other related analysis supported the candidate selection. The module containing *VvWRKY47* showed strong correlations with ROS markers and antioxidant enzyme activities. Furthermore, heterologous overexpression of *VvWRKY47* in the model plant *Arabidopsis thaliana* significantly enhanced its tolerance to abiotic stress, providing functional validation for our hypothesis. The WRKY family has also been incompletely studied in grapevine, and only a few members have been characterized (Vodiasova et al., 2024). For example, functional characterization of *VhWRKY44* showed stronger resistance to salt and cold stress in *Arabidopsis thaliana* (Zhang et al., 2024). A study related to biotic stress also showed that overexpression of *VqWRKY31* enhances disease resistance in grapevine (Yin et al., 2022). Further experiments are required to precisely identify the mode of regulation in grapevine. Additional research on such candidate genes in grapevine can validate their role in grapevine defense mechanisms. Further functional characterization of WRKY47 will be essential to elucidate its regulatory mechanism and validate its potential for enhancing saline-alkali stress resilience in grapevine breeding programs.

There are some limitations in our study. The experiment was conducted under controlled greenhouse conditions with potted plants, which is not fully same as the conditions in vineyard. In addition, functional validation of *VvWRKY47* was performed in Arabidopsis rather than grapevine; stable transformation or transient overexpression in *Vitis* will be essential to confirm its regulatory function in the native context.

## 5 Conclusion

This study shows that ‘Crimson Seedless’ grapevine responds to combined saline-alkali stress through physiological and transcriptional reprogramming. Transcriptomic analyses revealed coordinated enrichment of flavonoid biosynthesis, phenylpropanoid metabolism, and plant hormone signaling as central adaptive strategies. WGCNA identified *VvWRKY47* as candidate TF gene bridging stress signal and downstream antioxidant responses. Heterologous overexpression in Arabidopsis provided preliminary functional evidence supporting a role of *VvWRKY47* in stress tolerance. Collectively, these findings advance our mechanistic understanding of grapevine saline-alkali adaptation and identify *VvWRKY47* and associated metabolic pathways as priority targets for molecular breeding and biotechnological improvement of stress tolerance in commercially important *Vitis* cultivars.

## Data Availability

The datasets presented in this study can be found in online repositories. The names of the repository/repositories and accession number(s) can be found below: NCBI BioProject, PRJNA1499876.
