# Supplementary material for: Temporal dynamics of photosynthetic inhibition, antioxidant defense, and transcriptomic reprogramming in ‘Crimson Seedless’ grapevine under saline-alkali stress
Source: Front Plant Sci. 2026 Sep 24;17:1951854. doi: 10.3389/fpls.2026.1951854 (PMC13648038; doi:10.3389/fpls.2026.1951854)
Supplement: Supplementary file 1 [file DataSheet1.docx]

Figure S1. Correlation coefficient plot comparing RNA-seq and qRT-PCR fold changes.
